# Feeding rumen-protected choline to prepartum Holstein cows in negative energy balance increases circulating lipoprotein phosphatidylcholine and triglyceride concentrations

**DOI:** 10.3168/jdsc.2024-0661

**Published:** 2025-03-03

**Authors:** W.A. Myers, M.G. Zenobi, D.C. Reyes, J.E.P. Santos, C.R. Staples, J.W. McFadden

**Affiliations:** 1 Department of Animal Science, Cornell University, Ithaca, NY 14853; 2 Department of Animal Sciences, University of Florida, Gainesville, FL 32611; 3 Balchem Corp., Montvale, NJ 07645; 4 Department of Animal Nutrition, Faculty of Agricultural Sciences, National University of Córdoba, Córdoba 5000, Argentina

## Abstract

Rumen-protected choline (RPC) supplementation in dairy cows may increase hepatic phosphatidylcholine (PC) synthesis, thereby promoting triacylglycerol (TAG) secretion within very-low-density lipoproteins. To investigate the effects of dietary RPC on lipoprotein PC and TAG concentrations, pregnant, nonlactating, multiparous Holstein cows were supplemented with RPC (ReaShure, Balchem Corp.) at 0 (control), 30, 60, 90, and 120 g/d, corresponding to choline ion equivalents of 0, 6.45, 12.9, 19.4, and 25.0 g/d, respectively. Cows were fed diets that met or slightly exceeded their net energy and MP requirements for maintenance and pregnancy for 5 d, followed by a 9-d period of restricted intake (∼31% of these requirements). Preprandial plasma (n = 41, from cows in 0, 60, and 120 g/d RPC treatments) and liver samples (n = 72, from cows across all RPC treatments) were collected on d 9 of feed restriction. Plasma TAG-rich and low-density lipoprotein (LDL) fractions were isolated using liquid chromatography. Total TAG, cholesterol, and phospholipid concentrations in these lipoprotein fractions were measured. Circulating lipoprotein fractions and liver samples were processed for lipidomics. Data were analyzed using a mixed model, with calf birth weight and days prepartum at enrollment as covariates. Normalized omics data were natural-log-transformed. Plasma concentrations of total TAG within the TAG-rich lipoprotein fraction increased both linearly and quadratically, whereas total cholesterol exhibited a linear increase with higher RPC intake. In the LDL fraction, total TAG concentrations showed a similar linear and quadratic response, whereas total phospholipid concentrations increased linearly, and cholesterol concentrations remained unaffected by RPC supplementation. Most detectable PC and ether-linked PC species concentrations within the TAG-rich lipoprotein fraction increased linearly with RPC intake (42 out of 45 species; e.g., PC 34:3). Conversely, dietary RPC supplementation resulted in a modest increase in hepatic concentrations of PC species compared with the control (36 out of 57 species), characterized by an increase in highly unsaturated species (e.g., 36:5) and a decrease in those with a lower degree of saturation. Furthermore, increasing amounts of supplemental RPC resulted to linear, quadratic, and overall increases in the hepatic ratio of select PC to PE ion intensities compared with the control (8, 11, and 13 out of 17, respectively). In conclusion, RPC supplementation increased TAG concentrations within lipoprotein fractions in Holstein cows experiencing negative energy balance. The observed changes in hepatic and lipoprotein PC concentrations support enhanced very-low-density lipoprotein secretion in cows fed RPC. These findings suggest that RPC increases lipoprotein PC and TAG concentrations and reduces hepatic TAG deposition in dairy cows.

## Highlights

• Choline supplementation increased plasma lipoprotein triacylglycerol in cows.
• Cows fed choline had increased lipoprotein PC concentrations.
• Choline increased hepatic PC to phosphatidylethanolamine (PE) ratios.

## Graphical Abstract

**Figure:**
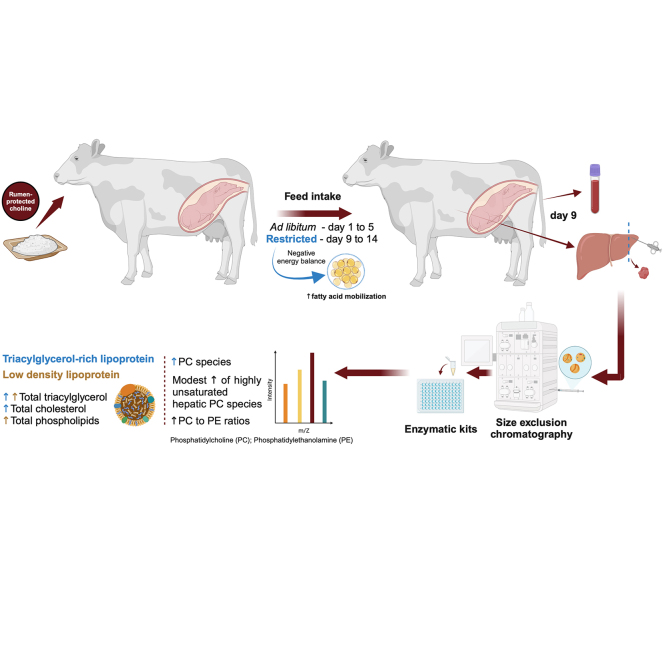
**Summary:** Limited hepatic export of triacylglycerol within lipoproteins can contribute to fatty liver disease in transition dairy cows. Dietary supplementation with choline may enhance the hepatic synthesis of phosphatidylcholine (PC), which is required to assemble and process very-low-density lipoproteins. We evaluated the effects of feeding rumen-protected choline on lipoprotein triacylglycerol and phospholipid concentrations in nonlactating, pregnant Holstein cows experiencing negative energy balance. Our findings suggest that rumenprotected choline supplementation increases lipoprotein triacylglycerol concentrations and modifies hepatic PC concentrations, potentially improving liver health in prepartum dairy cows.

Fatty liver disease affects ∼50% of dairy cows during the transition from gestation to lactation (Bobe et al., 2004). Despite extensive research spanning 3 decades, it remains a concern for the dairy industry (Wensing et al., 1997; D'Occhio et al., 2019). This syndrome is associated with decreased milk production, infertility, and negative health outcomes (Gaal et al., 1983; Wensing et al., 1997). The underlying cause of fatty liver disease is attributed to a voluntary reduction in DMI and increased energy demand at the onset of lactation (Drackley et al., 2001). As a result, an excessive mobilization of fatty acids from adipose tissue occurs, with a fraction of the mobilized fatty acids taking up and accumulating in the liver as re-esterified triacylglycerol (**TAG**; Bobe et al., 2004).

Phosphatidylcholine (**PC**), a glycerophospholipid, is required for assembling very-low-density lipoproteins (**VLDL**), which export TAG out of the liver (Grummer, 1993; Li and Vance, 2008). During the transition period, the concentrations of PC decrease in plasma and milk but increase as lactation progresses (Baldi and Pinotti, 2006; Artegoitia et al., 2014). Interestingly, ruminants may have a lower capacity to export hepatic TAG via VLDL compared with nonruminants (Kleppe et al., 1988). Hepatic PC synthesis involves 2 major pathways: (1) the cytidine diphospho-choline pathway, which relies on choline as a substrate, and (2) the phosphatidylethanolamine (**PE**) *N*-methyltransferase (**PEMT**) pathway, which requires ethanolamine and methyl groups provided by Met, betaine, or choline (DeLong et al., 1999). In rodents, these pathways contribute 70% and 30% of total hepatic PC production, respectively (Kennedy and Weiss, 1956). However, data from sheep and lactating goats suggest that the cytidine diphospho-choline pathway is of limited importance in ruminants (Dawson et al., 1981; Emmanuel and Kennelly, 1984), likely because of the extensive ruminal degradation of choline (Sharma and Erdman, 1988).

Supplementing dairy diets with rumen-protected choline (**RPC**) is a potential strategy to improve liver health and milk production in transition cows (Grummer, 2008; Arshad et al., 2020). However, the effectiveness of RPC supplementation in reducing liver TAG has been inconsistent due to variations in feeding duration, choline ion concentration, and the degree of ruminal protection (Piepenbrink and Overton, 2003; Zhou et al., 2016). In previous research, we observed a linear decrease in liver TAG concentrations with increasing RPC levels (up to 25.8 g of choline ion per day) in cows experiencing negative energy balance (Zenobi et al., 2018; Arshad et al., 2023a). However, the specific effects of RPC on species within lipoprotein fractions remain unclear. Therefore, we aimed to determine whether dietary RPC supplementation increases lipoprotein TAG and PC concentrations or modifies the composition of lipid species in these fractions in pregnant, nonlactating, multiparous Holstein cows experiencing negative energy balance. We hypothesized that increasing RPC levels would improve lipid metabolism by increasing plasma TAG and PC concentrations in lipoprotein fractions while reducing hepatic TAG accumulation, potentially mitigating the risk of fatty liver disease.

A total of 77 pregnant, nonlactating, multiparous Holstein cows averaging (± SD) 64 ± 10 d prepartum, BCS 3.59 ± 0.33, and 732 ± 96 kg of BW were used in a randomized complete block design experiment conducted at the University of Florida dairy farm (Gainesville, FL). Cows were assigned to 1 of 9 blocks according to BCS, as detailed by Zenobi et al. (2018). Within each block, cows were randomly allocated to 1 of 5 top-dressed amounts of RPC (ReaShure, Balchem Corporation, New Hampton, NY): 0, 30, 60, 90, and 120 g/d providing 0, 6.45, 12.9, 19.4, and 25.8 g/d of choline ions, respectively. Cows were fed a corn silage-based TMR once daily at 0900 h, formulated to meet or exceed their NEL and MP requirements during a 5-d ad libitum period. Uniform metabolic baseline conditions were established during this phase, with no differences in blood metabolites or liver TAG concentrations observed across treatments (Zenobi et al., 2018). Following this period, cows were subjected to a 9-d feed restriction (∼31% of their NEL and MP requirements) to induce negative energy balance, promoting fatty acid mobilization from adipose tissue and increasing liver TAG esterification and deposition (Cooke et al., 2007). Energy balance, calculated using NRC (2001) equations as outlined by Zenobi et al. (2018), averaged (± SD) −9.25 ± 1.12 and −9.16 ± 1.13 Mcal NEL/d for cows included in blood (n = 41) and liver (n = 72) assays, respectively. The RPC supplement was mixed with ground corn, molasses, and salt and top-dressed onto the TMR daily.

Blood samples were collected from 41 cows across 3 treatments (0, 12.9, and 25.8 g/d of choline ions) via preprandial coccygeal venipuncture on d 9 of feed restriction, using K_2_-EDTA-treated tubes (BD Vacutainer; Becton, Dickinson and Co.). Samples were centrifuged within 30 min of harvesting at 1,125 × *g* at 4°C for 15 min and snap-frozen at −80°C until analysis. Liver samples were collected from 72 cows across all 5 treatments (0, 6.45, 12.9, 19.4, and 25.8 g/d of choline ions) preprandially on d 9 of feed restriction. Samples were obtained using a steel percutaneous liver biopsy instrument (Aries Surgical) between the 11th and 13th rib intercostal space, guided by ultrasonography (Aloka SSD-500V with a 3.5-MHz convex transducer, Aloka Co. Ltd.). Liver samples were rinsed with saline, sectioned thrice, and immediately snap-frozen in liquid nitrogen before storage at –80°C until further analysis.

Size exclusion chromatography-fast protein liquid chromatography was used to separate plasma TAG-rich lipoprotein fractions containing VLDL and chylomicron remnants, and low-density lipoprotein (**LDL**) fractions. A BioLogic Duoflow-F10 and BioFrac collection systems were paired with a Superdex 650 10 × 300 mm column (Bio-Rad Laboratories). A phosphate buffer solution was prepared and used as an eluent buffer as described by Phipps et al. (2017). The solution was vacuum filtered through a 0.1-μm GP Express PLUS membrane (EMD Millipore). The buffer was degassed under vacuum and stirred with a sterile stir bar for 30 min. Plasma samples were thawed, centrifuged (10,000 × *g*, 4°C, 5 min), and injected (350 μL) with a 1-mL phosphate buffer solution. The buffer flowed at 0.3 mL/min, reaching 820 kPa, with absorbance measured continuously at 280 nm. Lipoprotein fractions (0.5 mL) were collected in 1.5-mL polypropylene tubes pretreated with 0.1% Tween 20 (Sigma-Aldrich). Fractions were identified by eluted peaks, pooled, and stored at −80°C. Fractionated TAG-rich and low-density lipoproteins were analyzed for total phospholipid, cholesterol, and TAG content using enzymatic kits (Phospholipid C #997-01801 and Cholesterol E #999-02601, Wako Chemicals USA Inc.; Triglyceride #T7532, Pointe Scientific Inc., respectively). Intra- and inter-assay CV were 1.27% and 3.0%, 1.63% and 1.28%, 0.97% and 1.07%, respectively.

Lipoprotein variables were analyzed using the MIXED procedure of SAS 9.4 (SAS Institute Inc.). The model included treatment (0 vs. 12.9 vs. 25.8 g/d or 0 vs. 6.45 vs. 12.9 vs. 19.4 vs. 25.8 g/d) as fixed effect and block as random effect, with calf BW and days from cow enrollment to calving as covariates. The Kenward-Roger method estimated denominator df for the *F* tests. For plasma analyses, linear and quadratic effects of choline ion intake (0–25.8 g/d) were tested using 2 orthogonal contrasts. For liver analyses, 4 contrasts tested linear, quadratic, and cubic effects of choline ion intake (0–25.8 g/d), plus a control (0 g/d) versus all RPC treatments. Significance was declared at *P* < 0.05 and tendencies at 0.05 ≤ *P* ≤ 0.10.

The concentrations of total TAG within the TAG-rich lipoprotein fraction increased both linearly and quadratically (*P* = 0.01) with increasing RPC supplementation (Table 1). Additionally, we observed a linear increase (*P* = 0.01) in total cholesterol, whereas total phospholipid content tended (*P* = 0.09) to increase quadratically (Table 1). Within the LDL fraction, total TAG concentrations increased linearly (*P* = 0.02) and quadratically (*P* = 0.04), total phospholipid concentrations showed a linear increase (*P* = 0.01), and total cholesterol tended (*P* = 0.09) to increase linearly with increasing amounts of RPC (Table 1).

**Table 1:** Plasma concentrations of total triacylglycerol (TAG), cholesterol, and phospholipids in TAG-rich and low-density lipoprotein fractions in response to dietary rumen-protected choline (RPC) supplementation (0 to 120 g/d) in pregnant, nonlactating Holstein dairy cows (n = 41) experiencing negative energy balance^1^

Metabolite, mg/dL	RPC dose,^2^ g/d			SEM	*P*-value^3^	
	0	60	120		Linear	Quadratic
TAG-rich lipoprotein						
TAG	0.97	2.03	1.77	0.22	0.01	0.01
Cholesterol^4^	0.65	1.04	1.11	0.13	0.01	0.21
Phospholipids	1.53	1.76	1.66	0.28	0.26	0.09
Low-density lipoprotein						
TAG	0.74	0.96	0.92	0.08	0.02	0.04
Cholesterol^4^	34.0	38.5	41.2	3.10	0.09	0.80
Phospholipids	15.9	19.4	21.6	1.38	0.01	0.69

1 Mean (± SD) energy balance was −9.25 ± 1.12 Mcal NEL/d.

2 Supplementation with 0, 60, and 120 g/d of RPC (ReaShure, Balchem Corp.) provided 0, 12.9, and 25.8 g/d of choline ion (n = 13, 13, and 15 cows), respectively.

3 Probability of linear and quadratic effects of supplementing increasing amounts of RPC. Significance was declared at *P* < 0.05 and tendencies at 0.05 ≤ *P* ≤ 0.10.

4 Free and esterified cholesterol.

The observed increase in total TAG concentrations within lipoprotein fractions following RPC supplementation suggests enhanced TAG export from the liver as VLDL. This is further supported by the linear increase in total plasma cholesterol concentrations within TAG-rich fractions, which increased linearly with RPC supplementation, suggesting enhanced VLDL production, as cholesterol is a primary constituent of these lipoproteins. Additionally, the phospholipid content in TAG-rich lipoproteins tended to increase, likely due to enhanced choline supply to the liver supporting PC synthesis, which is essential for VLDL formation and efficient lipid transport (Yao and Vance, 1990). Interestingly, rats supplemented with choline also exhibited increased plasma phospholipid concentrations compared with choline-deficient rats (Lombardi et al., 1966). Overall, our findings suggest that RPC supplementation promotes increased hepatic VLDL secretion. This is consistent with previous in vitro and in vivo research demonstrating the role of choline and RPC in increasing hepatic TAG secretion (Chandler and White, 2017; Arshad et al., 2023b,c).

The relative ion intensities of most detectable PC and ether-linked PC (**PC-O**) species within the TAG-rich lipoprotein fraction in both plasma and liver increased (*P* < 0.05) with RPC supplementation. In plasma, a linear increase was observed for 42 out of 45 species (e.g., PC 34:1, 36:1, 36:3, 38:3, and 38:5). Compared with the control (0 g/d), the mean response across all RPC levels showed increased ion intensities for 44 out of 45 species (Figure 1A and 1B). In the liver, RPC supplementation increased (*P* < 0.05) the ion intensities of 36 out of 57 species relative to the control (Figure 1C and 1D). Hepatic total PC species showed a modest increase in ion intensities with RPC supplementation, increasing from 59.6 to 62.1 ± 2.2 (mean ± pooled SEM) at 0 and 120 g/d, respectively (Figure 1E). Among these, the highly unsaturated species PC 36:5 exhibited a linear increase (*P* = 0.03) from 1.39 to 1.53 ± 0.05 at 0 and 120 g/d of RPC (Figure 1G). In contrast, total plasma TAG-rich lipoprotein PC showed a more pronounced response to RPC supplementation, increasing from 9.03 to 12.5 ± 0.72 at 0 and 120 g/d, respectively (Figure 1F). The ion intensities of the unsaturated species PC 34:3 showed both a linear (*P* < 0.01) and cubic response (*P* < 0.01), with values increasing from 0.27 to 0.34 and 0.95 ± 0.05 for 0, 60, and 120 g/d of RPC, respectively (Figure 1H).

**Figure 1:**
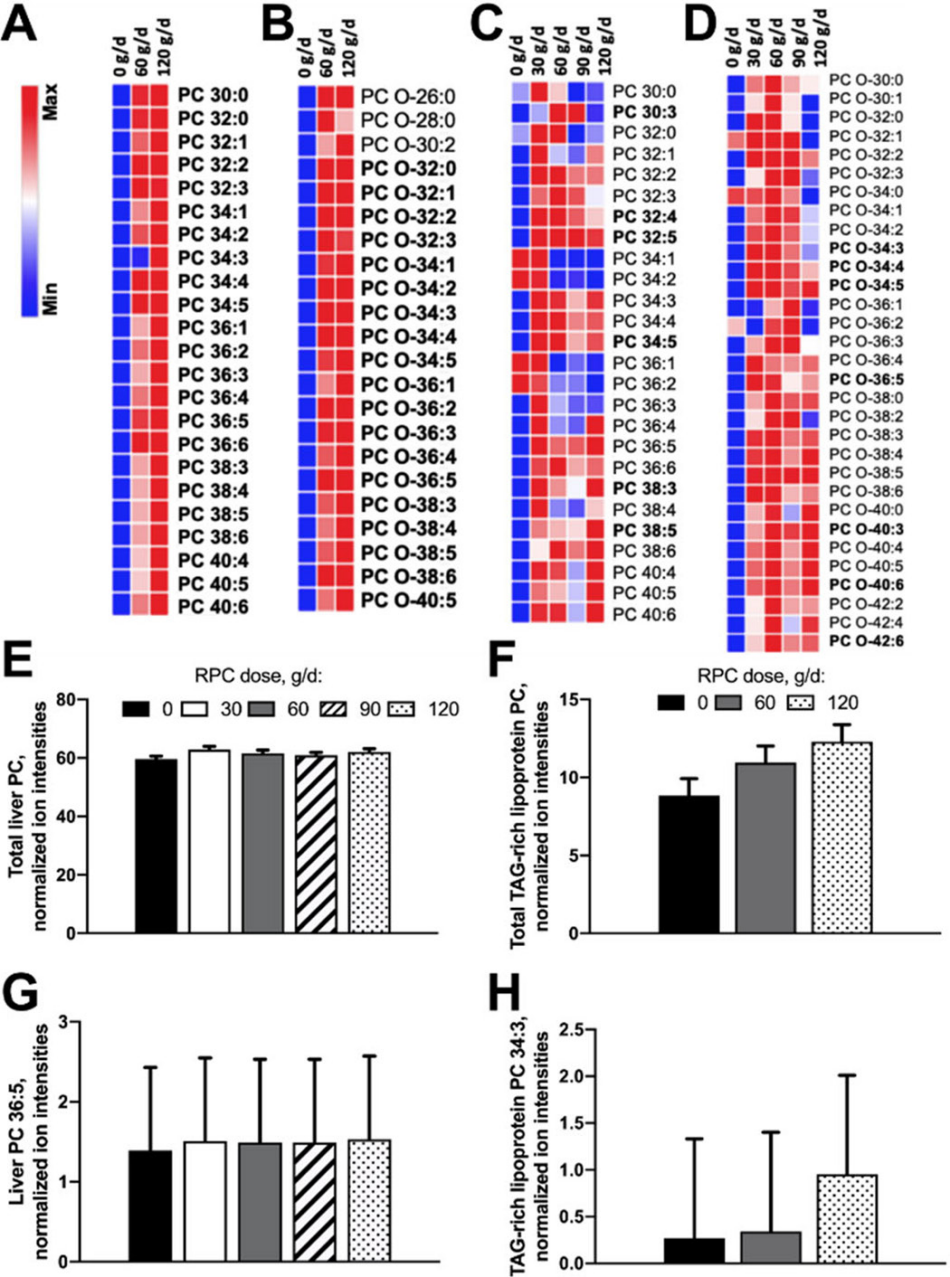
Fold change in relative ion intensities of phosphatidylcholine (PC) and ether-linked PC (PC-O) species in TAG-rich lipoprotein fraction of plasma (n = 41; A and B) and liver (n = 72; C and D), and normalized ion intensities of total PC species in liver (n = 72; E) and plasma TAG-rich lipoproteins (n = 41; F), and individual PC species 36:5 in liver (n = 72; G) and 34:3 in plasma TAG-rich lipoproteins (n = 41; H), in pregnant, nonlactating Holstein dairy cows experiencing negative energy balance supplemented daily with rumen-protected choline (RPC; ReaShure, Balchem Corp.) from 0 to 120 g/d. Error bars in panels E to H represent SEM. The heat map represents the relative fold change in ion intensity, with a blue-to-red gradient indicating low-to-high levels within each row. It includes 45 detectable PC and PC-O species, 42 of which increased linearly with RPC supplementation relative to control (*P* < 0.05). Species with varying chain lengths are shown in bold when overall statistically significant (*P* < 0.05).

Overall, TAG-rich lipoprotein PC and PC-O showed the largest increases with higher RPC supplementation. Our findings corroborate those of Zenobi et al. (2018), who reported a linear increase in plasma PC concentrations (e.g., 16:0/18:1, 16:0/20:3, 18:0/18:1, 18:0/20:3, and 18:0/20:5) with increasing RPC doses during the restricted feeding period (Zenobi et al., 2018). The comparatively modest increase observed in liver PC concentrations, relative to TAG-rich lipoprotein PC, suggests that most measured PC were actively transported within VLDL. In the current study, we observed slight increases in the concentrations of PE and ether-linked PE (**PE-O**) species within TAG-rich lipoprotein with RPC supplementation, particularly for those containing longer carbon chain lengths and more double bonds (e.g., PE 38:5; data not shown). This suggests these species may originate from the PEMT pathway, which produces highly unsaturated PE (DeLong et al., 1999). In contrast, hepatic PE and PE-O species were largely unaffected by RPC supplementation, except for a decrease in PE 36:2. This indicates that these species did not accumulate in the liver and may have been actively exported within TAG-rich lipoproteins, where modest increases were observed (data not shown). Interestingly, the few PE and PE-O species that increased in TAG-rich lipoproteins did not show corresponding decreases in their concentrations within hepatic tissue.

Dietary RPC supplementation increased the hepatic ratio of select PC to PE ion intensities, with 8 out of 17 exhibiting linear increases (*P* ≤ 0.05), 11 out of 17 quadratic trends (*P* ≤ 0.05), and 13 out of 17 increasing with RPC supplementation compared with the control (*P* ≤ 0.02; Table 2). As discussed above, reduced concentrations of hepatic PE indicate increased PEMT activity, potentially resulting in reduced VLDL PE concentrations and higher PC/PE ratios (Martínez-Uña et al., 2015). In nonruminants, dysregulation of the PEMT pathway and inadequate dietary choline intake are believed to contribute significantly to decreases in PC/PE ratios (Arendt et al., 2013). Furthermore, reduced hepatic PC/PE ratios can lead to hepatic steatosis in mice due to compromised cellular membrane integrity, which may contribute to the progression of steatohepatitis (Wan et al., 2019). Similar observations have been noted in humans with decreased hepatic PC/PE ratios and nonalcoholic steatohepatitis (Li et al., 2006). In our study, most hepatic PC/PE and PC-O/PE-O ratios showed negative correlations with liver TAG content and positive correlations with liver glycogen concentrations (data not shown). These findings further suggest that RPC supplementation may reduce liver TAG concentrations and increase hepatic gluconeogenesis or tissue glucose availability (Zenobi et al., 2018; Arshad et al., 2023b).

**Table 2:** Hepatic phosphatidylcholine (PC) to phosphatidylethanolamine (PE) species ratio of relative ion intensities in response to the dietary rumen-protected choline (RPC) supplementation (0 to 120 g/d) in pregnant nonlactating Holstein dairy cows (n = 72) experiencing negative energy balance^1^

Ratio of PC to PE	RPC dose,^2^ g/d					SEM	*P*-value^3^			
	0	30	60	90	120		Linear	Quadratic	Cubic	0 vs. rest
32:2	2.15	2.18	2.17	2.22	2.14	0.06	0.94	0.48	0.62	0.71
34:1	1.39	1.35	1.37	1.37	1.31	0.05	0.33	0.75	0.48	0.46
34:2	1.29	1.36	1.34	1.34	1.31	0.05	0.79	0.26	0.70	0.32
34:3	1.37	1.48	1.51	1.49	1.47	0.04	0.07	0.03	0.56	0.01
34:5	2.27	2.58	2.64	2.55	2.50	0.07	0.03	<0.01	0.15	<0.01
36:1	0.89	0.89	0.88	0.89	0.89	0.03	0.92	0.91	0.89	0.93
36:2	0.53	0.59	0.57	0.56	0.58	0.02	0.15	0.15	0.05	0.01
36:3	0.96	1.05	1.03	1.01	1.05	0.03	0.10	0.31	0.06	0.02
36:4	1.05	1.12	1.14	1.13	1.13	0.03	0.02	0.05	0.34	<0.01
36:6	1.55	1.71	1.75	1.71	1.69	0.05	0.05	0.01	0.36	<0.01
38:3	0.74	0.82	0.81	0.80	0.80	0.02	0.08	0.02	0.08	<0.01
38:4	0.40	0.42	0.44	0.42	0.42	0.01	0.22	0.01	0.37	0.01
38:5	0.66	0.73	0.74	0.74	0.75	0.02	<0.01	0.05	0.24	<0.01
38:6	0.71	0.77	0.82	0.82	0.79	0.02	<0.01	<0.01	0.90	<0.01
40:4	0.62	0.71	0.72	0.71	0.70	0.03	0.03	0.02	0.31	<0.01
40:5	0.69	0.77	0.79	0.79	0.77	0.03	0.03	0.01	0.48	<0.01
40:6	0.58	0.63	0.65	0.64	0.63	0.02	0.04	0.01	0.62	<0.01

1 Mean (± SD) energy balance was −9.16 ± 1.13 Mcal NEL/d.

2 Supplementation with 0, 30, 60, 90, and 120 g/d RPC (ReaShure, Balchem Corp.) provided 0, 6.45, 12.9, 19.4, and 25.8 g/d of choline ion (n = 14, 14, 14, 15, and 15 cows), respectively.

3 Probability of linear, quadratic, and cubic effects of supplementing increasing amounts of RPC and control (0) vs. all RPC treatment amounts. Significance was declared at *P* < 0.05 and tendencies at 0.05 ≤ *P* ≤ 0.10.

In conclusion, dietary RPC supplementation in pregnant dairy cows experiencing negative nutrient balance increased the concentrations of TAG and PC within circulating TAG-rich lipoprotein fractions. These results support the potential use of RPC as a nutritional strategy to reduce hepatic TAG accumulation by enhancing hepatic PC availability for VLDL synthesis in periparturient dairy cows. Although sampling on d 9 of feed restriction provided a limited temporal perspective on metabolic responses and the absence of baseline samples precluded direct comparisons to pretreatment conditions, our approach aimed to assess the cumulative effects of RPC supplementation during the restriction period. Furthermore, although apolipoprotein B_100_ concentrations and VLDL secretion were not directly measured, our findings, combined with those of Zenobi et al. (2018) and Arshad et al. (2023a,b,c), suggest that RPC supplementation may effectively decrease liver TAG deposition by promoting TAG export in dry pregnant dairy cows.
